# Characterization of the intergenerational impact of in utero and postnatal oxycodone exposure

**DOI:** 10.1038/s41398-020-01012-z

**Published:** 2020-09-23

**Authors:** Katherine E. Odegaard, Victoria L. Schaal, Alexander R. Clark, Sneh Koul, Austin Gowen, Jagadesan Sankarasubramani, Peng Xiao, Chittibabu Guda, Steven J. Lisco, Sowmya V. Yelamanchili, Gurudutt Pendyala

**Affiliations:** 1 Department of Anesthesiology, University of Nebraska Medical Center, Omaha, NE 68198 USA; 2 Department of Genetics, Cell Biology & Anatomy, University of Nebraska Medical Center, Omaha, NE 68198 USA

**Keywords:** Molecular neuroscience, Neuroscience

## Abstract

Prescription opioid abuse during and after pregnancy is a rising public health concern. While earlier studies have documented that offspring exposed to opioids in utero have impaired neurodevelopment, a significant knowledge gap remains in comparing the overall development between offspring exposed in utero and postnatally. Adding a layer of complexity is the role of heredity in the overall development of these exposed offspring. To fill in these important knowledge gaps, the current study uses a preclinical rat model mimicking oxycodone (oxy) exposure in utero (IUO) and postnatally (PNO) to investigate comparative and intergenerational effects in the two different treatment groups. While significant phenotypic attributes were observed with the two treatments and across the two generations, RNA sequencing revealed alterations in the expression of key synaptic genes in the two exposed groups in both generations. RNA sequencing and post validation of genes using RT-PCR highlighted the differential expression of several neuropeptides associated with the hypocretin system, a system recently implicated in addiction. Further, behavior studies revealed anxiety-like behaviors and social deficits that persisted even in the subsequent generations in the two treatment groups. To summarize, our study for the first time reveals a new line of investigation on the potential risks associated with oxy use during and after pregnancy, specifically the disruption of neurodevelopment and intergenerational impact on behavior.

## Introduction

The widespread abuse of prescription opioids and a dramatic increase in the availability of illicit opioids have created what is commonly referred to as the opioid epidemic^1^. As a particularly vulnerable group, pregnant women are prescribed opioids such as morphine, buprenorphine, and methadone to alleviate pregnancy and postpartum pain, all of which have been shown to cross the placenta^2–4^. Data collected by the Centers for Disease Control and Prevention from a 2008 to 2012 study found that more than a third of reproductive-aged women enrolled in Medicaid (39%) and more than a quarter of those with private insurance (28%) filled a prescription for an opioid pain medication each year^5^. Additionally, postpartum women, regardless of delivery method or pain measurement, commonly receive similar amounts of opioids upon discharge from the hospital^6,7^. The lack of standardization in prescribing patterns of these opioids contributes to a sizable amount of leftover medication, which can lead to nonmedical use of the prescriptions^6,8^. Among the prescription opioids, oxycodone (oxy) has recently emerged as a serious contender for widespread abuse. A postoperative analgesic, oxy has been reported in the literature for postpartum pain or caesarian sections in lieu of morphine drips^9,10^, potentially exposing neonates to this powerful opioid.

Several studies^11^ have been conducted with rodent models to investigate the detrimental effects of gestational opioid use on neurodevelopment of the offspring, but few of these studies consider oxy. Additionally, these prior studies relied on self-administration or continuous release pumps for drug administration, and very few have used oral delivery to mimic the usual route of administering pain medication. Orally-administered analgesics are the most common form of pain relief prescribed after caesarian sections^12,13^, and oral oxy administration has been shown to be safer than and as effective as intravenous administration of other opioids^9,14,15^. Previous oxy studies have shown deficits such as behavioral impairments and disruption in both *OPRM1* and endothelin receptor expression during development in offspring exposed to oxy in utero^16–19^. However, these studies focused primarily on prenatal oxy exposure, leaving a large gap in the knowledge regarding postnatal oxy exposure.

Opiates have been shown to pass into the placenta and act on fetal opioid receptors^2–4^. Opioids also accumulate in the breastmilk^20^. The degree of exposure of an infant to a drug passed through the breastmilk depends on the concentration of the drug in the milk, the amount of milk ingested, and the rate of elimination from the infant^21^. A human study by Seaton et al. showed that oxy is concentrated in the breastmilk, and offspring exposed via the breastmilk may receive <10% of a typical oral therapeutic infant dose (0.1–0.2 mg/kg)^22^. Despite this low dose, infant exposure to oxy via the breastmilk has been associated with sedation, central nervous system depression, and neonatal toxicity^23–26^, and a number of animal studies have also revealed deficits in behavior and development associated with perinatal opioid exposure^17–19,27^. The full extent of postnatal oxy exposure effects is not yet known particularly in regard to central neural synaptic function and gene expression. Further, pre- and postnatal opioid abuse can result in several phenotypic consequences across multiple generations of offspring, despite no previous exposure to drugs^28^. Currently, a gap in knowledge exists regarding the long-term, intergenerational effects of oxy exposure during the perinatal period on future generations.

The present study was performed to determine the intergenerational effects of in utero (IUO) and postnatal oxy (PNO) exposure on development in both the F1 and F2 generations. This study was conducted using a Sprague Dawley rat model our labs have previously established^29^. This model consists of two groups of pregnant F0 dams that had been orally-administered oxy. The first group was treated daily with an oral administration of oxy before, during, and after pregnancy, thereby exposing the F1 pups to IUO throughout fetal and postnatal development. The second group was treated daily with an oral administration of oxy only after giving birth, exposing the F1 pups to oxy postnatally. The F2 generation was descended from the germlines of F1 dams exposed to oxy via the breastmilk or in utero, allowing us to elucidate intergenerational effects. Importantly, both the PNO and IUO groups are clinically relevant^29^. The IUO dams represent women who exhibit chronic opioid use before, during, and after pregnancy, and the PNO group represents children exposed to opioids after their mothers are prescribed medication post-caesarian section. Additionally, the PNO group represents neonates in the neonatal intensive care unit (NICU) that may be exposed to high-dose opiates through infusions. Infants born with heart defects and persistent pulmonary hypertension are exposed to opiates for sedation and analgesia while being supported by extracorporeal membrane oxygenation (ECMO) and mechanical ventilation^30^. Infants may be supported by ECMO for 3 weeks or longer, exposing the newborns to potent opiates for an extended period of time and contributing to the increase in diagnoses of neonatal abstinence syndrome^31,32^. Little is known regarding the implications of these opiate drips and how they may impact neurodevelopment in newborns. Our PNO group provides a high-dose opiate exposure to the pups that is comparable to the high-dose opiate exposure neonates in the NICU experience, establishing clinical relevance for the inclusion of this group in future studies.

The present study uses a holistic integrated systems biology approach to determine the intergenerational effects of pre- and postnatal oxy exposure on development in both the F1 and F2 generations descended from oxy-exposed mothers. Our overall hypothesis was that F0 maternal oxy use and F1 in utero and postnatal oxy exposure result in developmental impairments (physical, molecular, and behavioral) in the F1 offspring that persist in the F2 generation. The comprehensive and systematic approach used in this study allows for thorough research into intergenerational effects of pre- and postnatal oxy abuse, a critical step in closing the knowledge gap surrounding this commonly used opioid analgesic.

## Methods

### Animals

Male and female Sprague Dawley rats were obtained from Charles River Laboratories Inc. (Wilmington, MA, USA) and group housed in a 12 h light–dark cycle and fed ad libitum. The complete number of animals used per group for each experiment can be found in Supplementary Table 1. All procedures and protocols were approved by the Institutional Animal Care and Use Committee of the University of Nebraska Medical Center and conducted in accordance with the National Institutes of Health Guide for the Care and Use of Laboratory Animals.

### Oxycodone treatment

The treatment paradigm was adapted from our lab’s previous work to include the F2 generation^29^. To elucidate intergenerational effects in the IUO and PNO groups, untreated F1 females (P70) from each condition were mated with male breeders naïve to the experiment (Supplementary Fig. 1). F2 pups were housed with their mothers until weaning (P21). All data presented are from complete litters per condition and include both male and female offspring.

### Phenotypic measurements

Body measurements included weight, body length, and head size circumference and were obtained from the complete litters at P1, P7, P14, and P30. Body mass index (BMI) and Lee’s Obesity Index (LOI) were calculated as described by Novelli et al.^33^.

### RNA-seq and post validation via RT-PCR

Total RNA from nucleus accumbens (NAc) tissue was isolated from the randomly selected male or female pups of each treatment group at P14 using the Direct-Zol RNA kit (Zymo Research, CA, USA). RNA samples were sent on dry ice to LC Sciences (Houston, TX, USA) for sequencing. Transcriptomes from all samples were merged to reconstruct a comprehensive transcriptome using a proprietary Perl script of LC Sciences (Houston, Texas, U.S.A.). Following transcriptome reconstruction, FPKM (Fragments Per Kilobase Million) reads were evaluated by StringTie, and differentially expressed genes (DEGs) were evaluated by edgeR. Raw data has been deposited to NCBI’s Gene Expression Omnibus (GEO; accession number GSE157348). Potential hits having ±1.5-fold expression and *p* < 0.05 were validated using relevant TaqMan probes by real-time PCR (RT-PCR). Further analysis into the Hcrt system was similarly done using RT-PCR TaqMan probes. Delta-delta Ct method^34^ was used to calculate fold change and statistical significance.

### Bioinformatic data analyses

Functional relevance of DEGs (up- and downregulated) was evaluated using the Cytoscape plug-in ClueGO^35^. A kappa score level threshold of 0.4 was used to restrict the GO network connectivity with three as minimum and eight as maximum level of the genes in each GO term.

### Behavioral studies

#### Social testing

Social testing consisting of social novelty and social preference were carried out in P60–65 F1 and F2 male and female rats from the different treatment groups using an in-house built chamber. Briefly, a 90 × 40 × 40 cm acrylic chamber was divided into three 30 × 40 × 40 cm compartments. Left and right compartments contained 15 × 15 × 30 cm isolation cubes with evenly-drilled holes spaced 1 cm apart along the entirety of the cube.

To evaluate social novelty, a naive animal of the same sex and of similar age and size was placed into the left isolation cube. A cagemate of the test animal’s housing cage was placed into the right isolation cube. The test animal was placed into the central chamber. For assessing social preference, a new naive rat (not used in social novelty) was placed into the left isolation cube, a rubber toy in the right isolation cube, and the test animal into the central chamber. After 5 min of acclimation, the two doors were lifted and the test animal was allowed to freely explore the entirety of the social chamber for 15 min. Animals were then returned to their housing cages, and the social chamber was cleaned and sterilized.

Scoring for both social tests consisted of the time the animal spent in each chamber, the number of entries into each chamber, and the number of active contacts toward one of the isolation cubes. Entry into a chamber was scored if an animal’s head and all four paws were within the compartment. An active contact was defined as any attempt to sniff, paw, scratch, touch, or stretch toward any of the isolation cubes when inside the compartment containing an isolation cube. Testing was recorded, and recordings were scored manually by scorers blinded to the conditions.

#### Marble burying

Marble burying was tested on all three groups on male and female pups between ages P65 and P70. A rat cage (929 cm^2^, 43.18 × 21.59 × 20.32 cm) contained a leveled 5 cm layer of ¼-inch corncob bedding (Envigo #7097), and 20 standard glass marbles (15 mm diameter, 5.2 g) were lightly placed in a 5 × 4 arrangement along the bedding. The subject was placed into the cage, and the cage was covered for 30 min. The animal was removed, and the marbles were imaged and scored by a scorer blinded to the conditions. A marble was considered buried if more than 2/3 of a marble was under the bedding.

### Data and statistical analyses

All data represented in the paper are reported as mean ± SEM. Data in each analysis were normally distributed. Significant intergenerational differences were computed using two-way ANOVA followed by Tukey’s test with a significance criterion of *p* ≤ 0.05. Main interactions were further analyzed independently within generations using one-way ANOVA followed by Tukey’s test with a significance criterion of *p* ≤ 0.05. Two-way ANOVA was also used for social testing analysis (row factor: chamber; column factor: treatment), followed by Tukey’s test. All variances are listed in Supplementary Statistical Data, sorted by figure number. Data were analyzed using the Graph Pad Prism software (La Jolla, CA, USA).

## Results

### Oxy induces intergenerational developmental differences in exposed pups

**Fig. 1:**
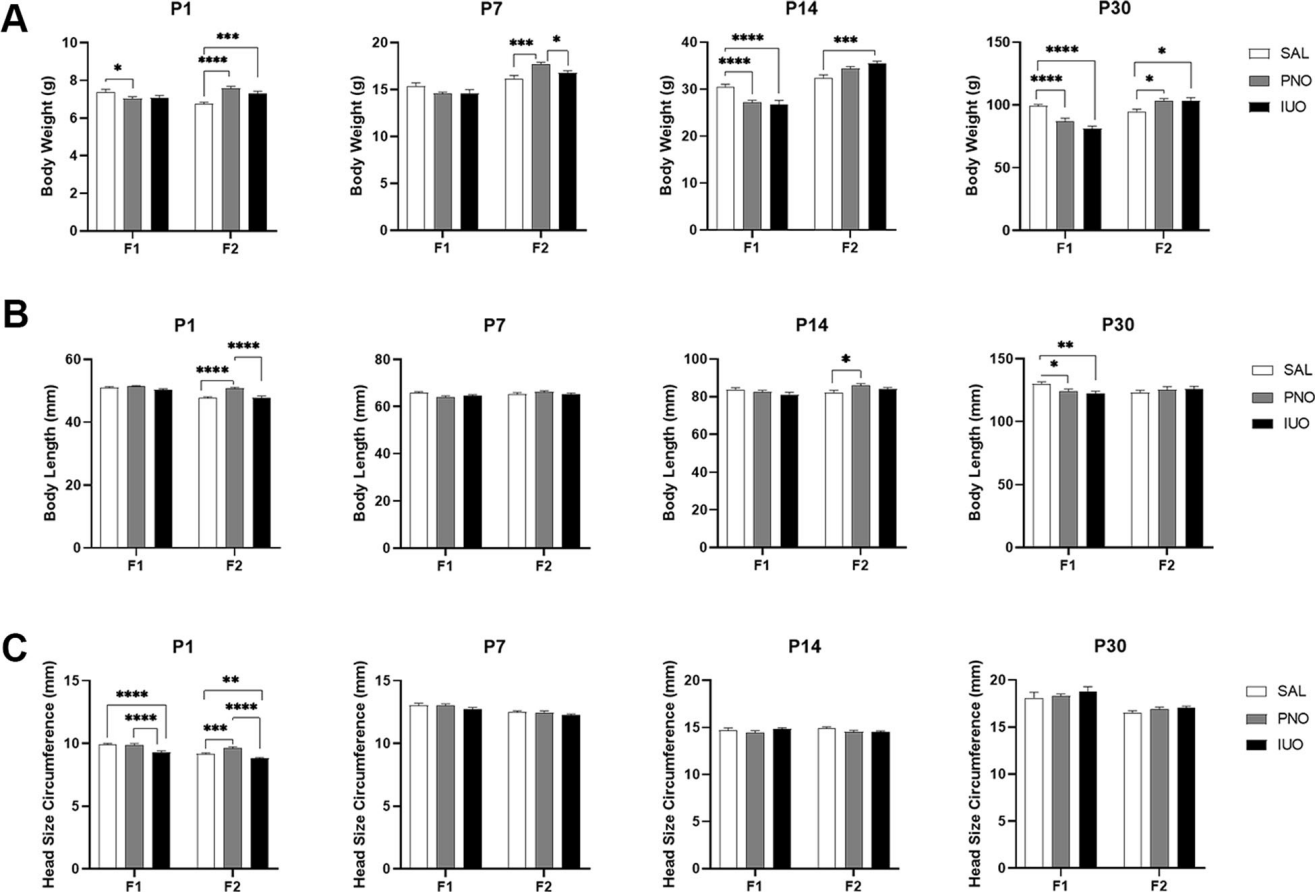
Phenotypic measurements. **a**–**c** Alterations in physical development patterns in both the IUO and PNO offspring as observed through body weight, body length, and head size circumference. *p < 0.05; ***p < 0.001; ****p < 0.0001 as determined by two-way ANOVA followed by a post hoc Tukey’s test.

**Fig. 2:**
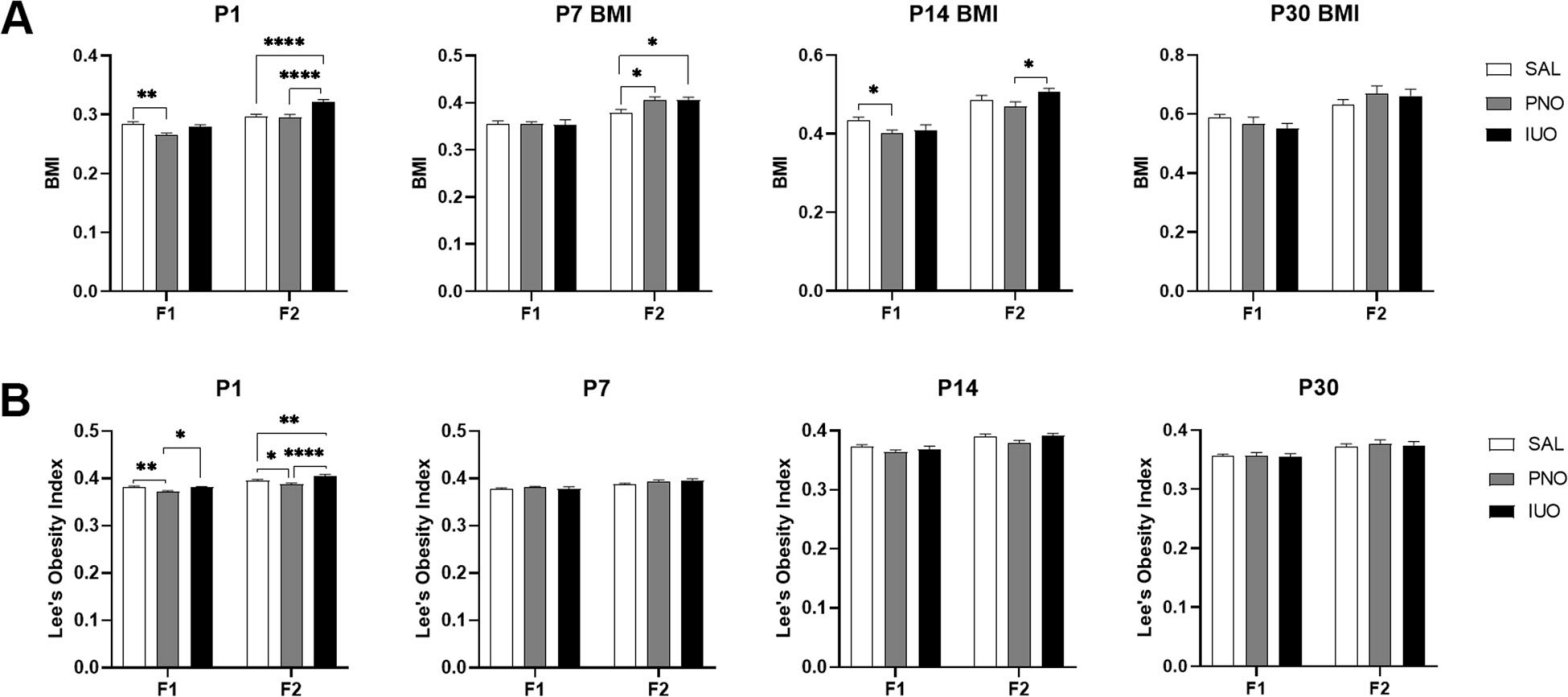
Phenotypic measurements. **a**, **b** Alterations in physical development patterns in both the IUO and PNO offspring as observed through body mass index (BMI) and Lee’s Obesity Index (LOI). *p < 0.05; **p < 0.01; ****p < 0.0001 as determined by two-way ANOVA followed by a post hoc Tukey’s test.

To determine whether oxy exposure affects physical development in the PNO and IUO pups, we measured head size, body weight, and body length on postnatal days (P) 1, 7, 14, and 30 in both generations (Fig. 1). Additionally, we calculated the BMI and LOI to estimate obesity and body fat, respectively (Fig. 2). By measuring at these time points, we can assess perinatal development through periadolescence^36^. Additionally, these time points correspond with human aging from infant, young child, childhood, and preadolescence^37^.

At P1 in the F1 animals, PNO pups weighed less than controls. While there were no differences in F1 weights at P7, a dramatic decline was observed in both PNO and IUO groups at P14 that persisted through P30. Interestingly, F2 PNO and IUO animals showed a significant increase in body weights compared to the saline controls at each time point (Fig. 1a). While no significant changes in body length were seen in the F1 pups at P1, P7, and P14, there was a marked reduction in the body length at P30. In the F2 pups, the PNO offspring had longer body lengths than both the saline and IUO groups at P1 and longer body lengths than controls at P14 (Fig. 1b). Head size circumference was markedly affected in the F1 and F2 IUO offspring at birth while F2 PNO pups had larger head size circumference than both IUO and controls at P1 (Fig. 1c).

BMI and LOI measurements were significantly different in the oxy-exposed groups of both generations. The F1 PNO group had both lower BMI (Fig. 2a) and a lower score on the LOI (Fig. 2b) than the saline and IUO groups at P1, suggesting this group may be underweight. Intriguingly, in the F2 animals, both the BMI (Fig. 2a) and LOI (Fig. 2b) indicated obesity and greater body fat in the IUO at P1. Additionally, while there were no differences in BMI in the F1 P7 animals, the F2 PNO and IUO groups had larger BMI than the controls at P7, indicating obesity (Fig. 2a). At P14, F1 PNO had a lower BMI than the saline controls while F2 IUO had a larger BMI than PNO (Fig. 2a). We did not observe any differences in BMI (Fig. 2a) in either generation at P30.

### RNA-seq identified distinct gene signatures and molecular pathways in the two generations

**Fig. 3:**
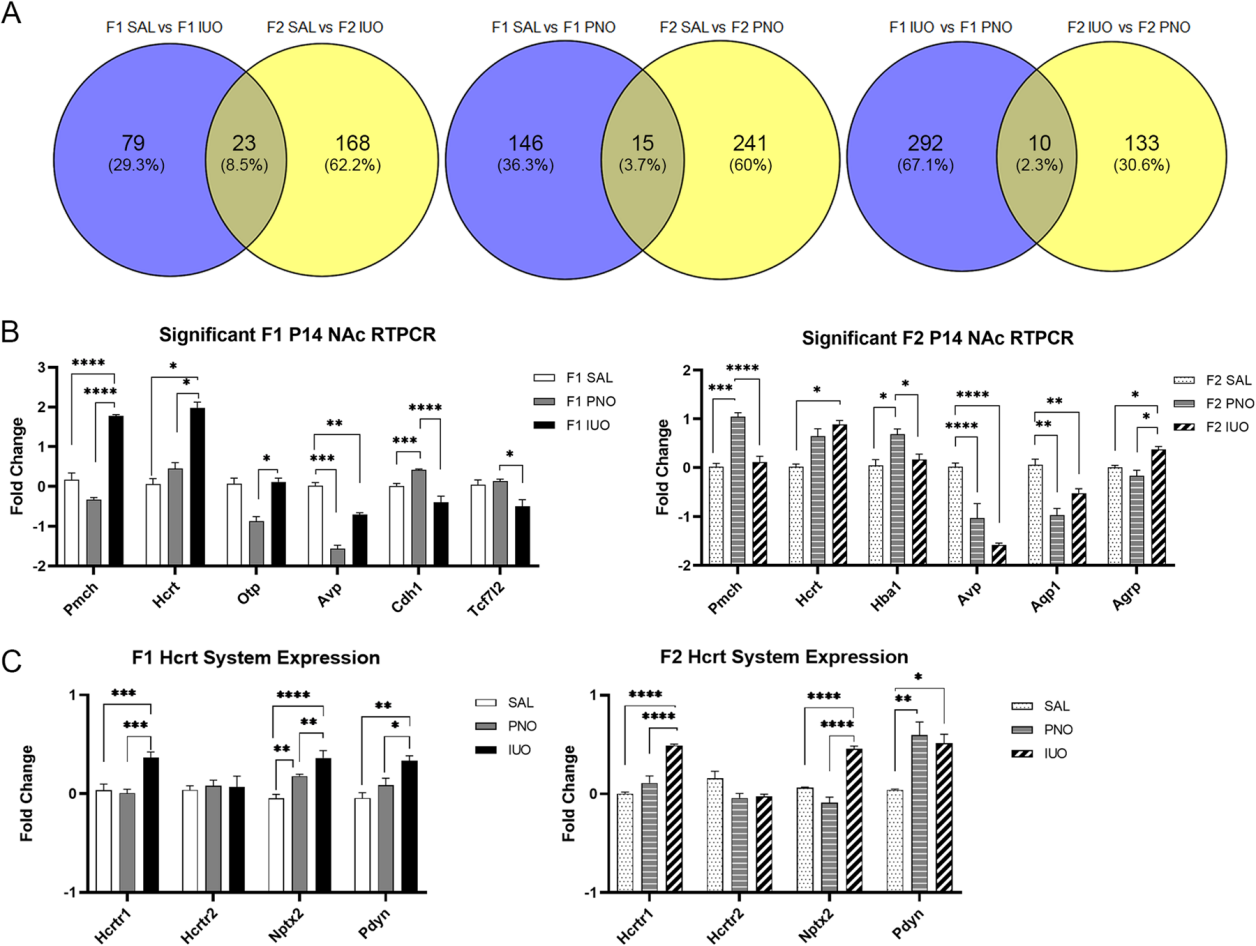
RNA-seq analysis on P14 nucleus accumbens (NAc) of F1 and F2 animals. **a** Venn diagram depicting the total number of genes affected in the comparison of treatment groups of each generation. **b** Of the genes post validated in the F1 NAc samples, *Pmch, Hcrt, Otp, Avp, Cdh1, Oxt*, and *Tcf7l2* were significant. Of the genes post validated in the F2 NAc samples, *Pmch, Hcrt, Hba1, Avp, Aqp1*, and *Agrp* were significant. *p < 0.05; **p < 0.01; ***p < 0.001; ****p < 0.0001 as determined by one-way ANOVA followed by a post hoc Tukey’s test. **c** Further investigation of the hypocretin system neuropeptide expression levels found *Hcrtr1*, *Nptx2*, and *Pdyn* were upregulated in the IUO group of both generations. *p < 0.05; **p < 0.01; ***p < 0.001; ****p < 0.0001 as determined by two-way ANOVA followed by a post hoc Tukey’s test.

Based on the significant physical attributes observed at P14 in the F1 and F2 generations, we performed RNA-Seq analysis on the NAc. The NAc was chosen given its association with the reward pathway. RNA-Seq analysis showed several up- and downregulated genes among the IUO, PNO, and saline groups in both the F1 and F2 generations (Supplementary Table 2). Using ClueGO analysis (Supplementary Fig. 2; Supplementary Table 3), we found that pathways involved in the regulation of neurological system process, forebrain and neural crest development, and synaptic transmission were affected by the DEGs in the F1 animals. In the F2 animals, we identified pathways involved in fear response, neurodevelopment, cell signaling, digestive processes, and heart rate regulation that were affected by the DEGs. Further, when comparing the same F1 group comparisons with the F2 comparisons, we found that a number of genes were consistently differentially regulated in both generations (Fig. 3a; Supplementary Tables 4–6). Of the differentially regulated genes in both the F1 and F2 generations of saline compared to IUO, 23 genes were common. For saline compared to PNO, 15 genes were commonly differentially regulated between the generations. Lastly, when comparing PNO and IUO, 10 genes were consistently differentially regulated in the F1 and F2 generations.

Potential hits based on a fold change value of ±1.5 that were involved in behavior and development included a total of 18 and 12 genes in the F1 and F2 generations, respectively (Supplementary Fig. 3). Post validation of these hits using RT-PCR resulted in six genes successfully post validated in the F1 and F2 generation (Fig. 3b). Interestingly, of the genes that were post validated in both F1 and F2, *Pmch, Hcrt*, and *Avp* were significant in both generations. *Hcrt* and *Avp* also had similar expression trends among the experimental groups in both F1 and F2. Intriguingly, the F1 IUO had significantly higher expression of *Pmch* compared to both groups, while the PNO had significantly higher expression in the same F2 comparison. These three genes are important in behavior and development and, based on their differential expression, may contribute to the differences observed between the F1 and F2 offspring.

Because the hypocretin (*Hcrt*) expression trends were the same in both generations, we sought to further investigate this system (Fig. 3c). We found that *Hcrtr1* expression was significantly higher in the IUO group in both generations. *Nptx2*, a protein expressed in orexin neurons, was also highly expressed in IUO compared to PNO and controls in both generations. In the F1 generation, the PNO also high a higher expression of *Nptx2* compared to controls. Further, *Pdyn*, the precursor for dynorphin (an inhibitory neuropeptide co-released with hypocretin), showed higher expression levels in IUO than both PNO and controls in the F1 generation; both IUO and PNO offspring had higher levels of *Pdyn* than controls in the F2 generation.

### Behavioral deficits continue in the next generation of oxy-exposed pups

Early life insults can significantly impact developmental and behavioral outcomes exhibited during adulthood. Based on our RNA-seq data that highlighted several genes associated with behavioral responses, we examined if oxy exposure induces intergenerational behavioral deficits during adulthood in both IUO and PNO pups. Accordingly, we performed social interaction and marble burying tests in adult animals (P60–70).

**Fig. 4:**
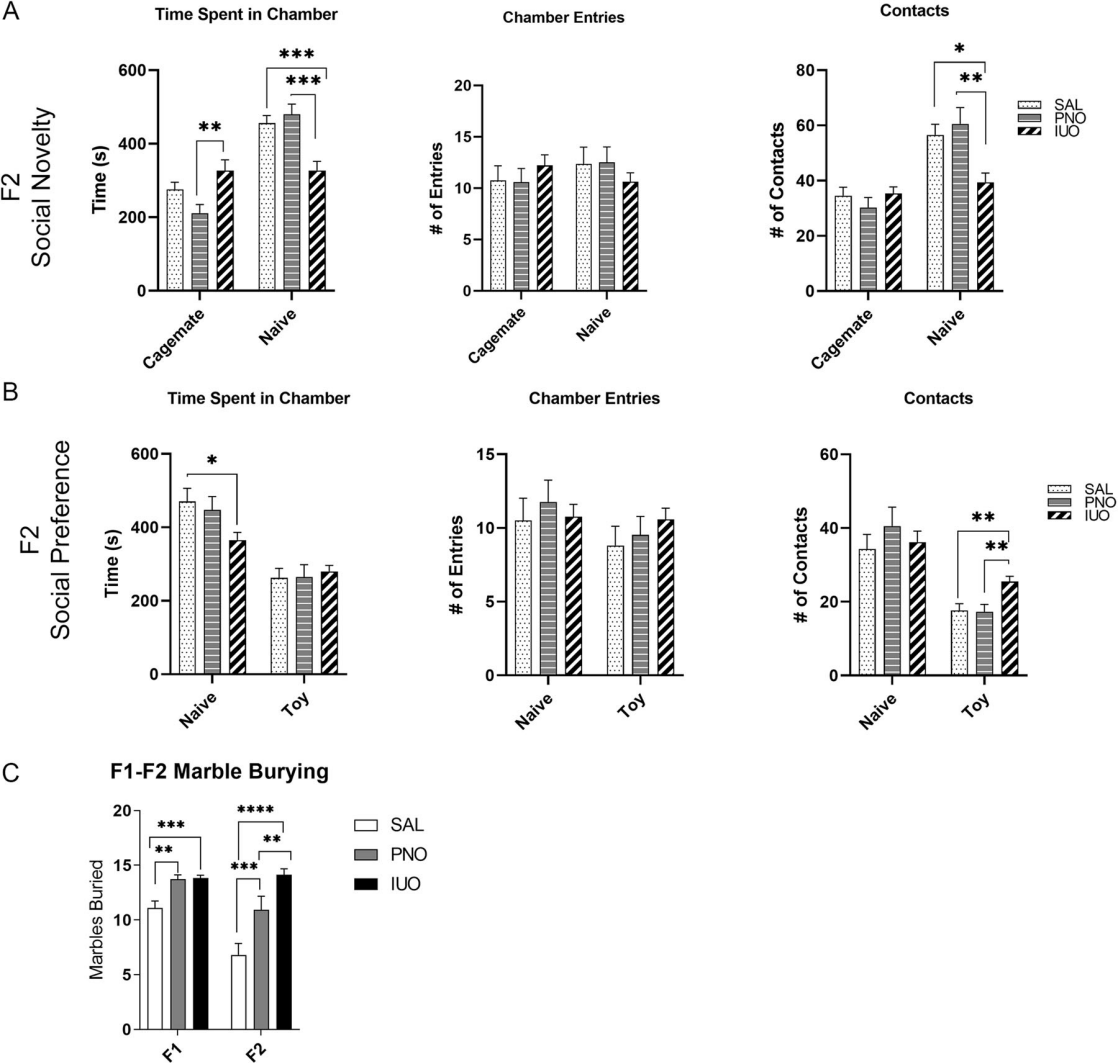
Behavior tests. **a** F2 social novelty testing revealed that IUO offspring spent less time with naive animals and more time with the cagemate than the other groups. IUO also had fewer contacts with the naïve animal. **b** F2 social preference testing showed IUO spent less time with the naive animal than the other groups, and IUO had more contacts with the toy than did the other groups. *p < 0.05; **p < 0.01; ***p < 0.001 as determined by two-way ANOVA followed by a post hoc Tukey’s test. **c** Marble burying tests in both generations showed increased burying activity in the PNO and IUO groups. **p < 0.01; ***p < 0.001; ****p < 0.0001 as determined by two-way ANOVA followed by a post hoc Tukey’s test.

In the F1 generation, social novelty tests revealed no significant differences in social interactions of the PNO and IUO groups (Supplementary Fig. 4). However, in the F2 generation, IUO offspring spent more time with the cagemate than the PNO group and less time with the naïve animal than both the control and PNO groups (Fig. 4a). Although not significant, the IUO demonstrated a higher tendency to enter the cagemate chamber than the other two groups (Fig. 4a). Additionally, when considering the number of contacts with the naïve animal, the IUO displayed fewer contacts with the naive animal (Fig. 4a), suggesting a social deficit and hesitancy to spend more time with or interact with the unknown animal. Similar to the F1 social novelty test results, F1 social preference tests revealed no significant differences in social interactions exhibited by the PNO and IUO groups (Supplementary Fig. 4). In the F2 social preference task, IUO animals spent less time with the naive animal than did either of the other groups (Fig. 4b), and IUO had more contacts with the toy than had either the PNO or control animals, further suggesting social deficits in the F2 generation.

Marble burying tests, which measure repetitive stereotypy, compulsive behaviors, and anxiety-like behavior^38^, showed that both the PNO and IUO groups in both generations buried more marbles than the controls, suggesting heightened anxiety and compulsivity in these animals (Fig. 4c). Interestingly, IUO animals in the F2 group buried more marbles than the PNO, suggesting that the F1 IUO exposure may result in more pronounced intergenerational anxiety and compulsive behaviors than F1 PNO exposure. In summary, in utero and postnatal oxy exposure can significantly alter behavioral outcomes that persist into the next generation.

## Discussion

The model system and dose of oxy (15 mg/kg) used in our study mimicked a chronic prescription opiate-dependent woman during gestation and parturition. This dose has been shown to be well-tolerated in animals and mimic development of chronic analgesia in human subjects experiencing breakthrough pain^39,40^. A gap in knowledge exists regarding the implications of how perinatal oxy exposure may impact neurodevelopment in newborns and affect long-term adult behaviors in an intergenerational manner. Intergenerational transmission of traits and phenotypes results from direct exposure of the F0 parent and F1 offspring to a stressor^41^, such as oxy. When the oxy exposure occurs in utero, as in the IUO group, the F1 fetus and its germlines are directly exposed. Additionally, when exposed via the breastmilk, which occurs in both the PNO and IUO groups, the germlines are also exposed. The exposure of the germ line within the F1 offspring results in an intergenerational transmission to the F2 generation. Studying the intergenerational effects of oxy exposure in utero and postnatally can highlight the long-term adverse consequences that extend beyond the mother into future generations.

Opioid use during pregnancy has been associated with smaller head sizes, lighter birthweights, and shorter body lengths in newborns^42–46^. Our data dovetail with this trend, showing that in utero oxy exposure affects birthweight, body length, and head development. A rat study using prenatal exposure to morphine yielded similar results, with offspring during the preweaning period having a lower body weight as well as a reduction in brain and cerebellar weights and widths^47^. Similarly, a study using buprenorphine showed delayed offspring development, decreased body weight, decreased body length, and lower pain sensitivity^48^. In our F1 generation, body length appeared to be delayed at P30 when compared to controls. Interestingly, the F2 PNO pups, specifically, had longer bodies at P1 and P14, but all F2 body lengths were comparable at P30. As for head size, decreased head size and body weight have been reported in a study of NICU neonates^49^. In our study, IUO head size circumference in both generations was smaller compared to controls at P1; head size circumference was comparable to controls at the remaining time points, however.

With regard to weight, in utero morphine exposure has also been shown to produce weight deficits that persisted in rats through adulthood^50^, much like what we observed in the F1 PNO and IUO groups. Eriksson and Ronnback showed that rats exposed to prenatal morphine treatment not only had lower birthweights than controls but also gained less weight than controls until P19^51^. In our study, F1 PNO and IUO pups appeared to maintain the weight deficit through P30 and did not approach weight levels comparable to saline controls. Our study also identified differences in BMI and LOI during early development. Intriguingly, IUO and PNO in the F2 generation had heavier body weights at all time points compared to controls. Additionally, while the F1 generation trended toward lower BMIs early in development, the F2 generation had higher BMI scores. In other studies considering underweight mothers and the weight gain of their offspring, maternal undernutrition improves the metabolic health of the next generation^52^ and can lead to weight gain and obesity in the next generation^53^, much like what we observed in our overweight F2 generation compared to their underweight F1 mothers.

After post validation of our RNA-seq results, the upregulation of *Hcrt* in the IUO groups of both generations led us to investigate the expression of key genes in the hypocretin system. Hypocretins are involved in arousal, but they are also involved in drug addiction and reward-related behaviors^54^. Hypocretin activation of Hcrtr1 is critical for morphine withdrawal, and NAc activation during withdrawal is dependent on Hcrtr1 function^55^. Not only do our data show an increase in *Hcrt* expression in IUO pups of both generations, which is associated with acute opiate withdrawal^56,57^, but the expression of *Hcrtr1* in the NAc is also increased significantly in the IUO, further suggesting these IUO pups may be experiencing acute withdrawal. Additionally, Nptx2, which regulates the clustering of AMPA receptors at the synapse, has been shown to increase following opiate withdrawal^58^. The IUO animals in both generations exhibited an increase in *Nptx2* expression, again suggesting withdrawal in these animals. Lastly, our IUO animals had higher levels of *Pdyn* expression in both generations. Chronic exposure to drugs of abuse results in the upregulation of the dynorphin (*Dyn*)/KOR system, and this system has been shown to contribute to psychiatric disorders such as anxiety, depression, and addiction^59,60^.

In addition to highlighting the hypocretin system, our RNA-seq data also highlighted several genes associated with behavioral responses; therefore we sought to investigate the impact of IUO and PNO exposure on social behaviors. In human studies, prenatal opioid exposure has been associated with increased social problems and difficulty with sociability^49,61^. In our social novelty and preference tasks, we did not observe any significant differences in social behaviors among the F1 groups; however, the F2 IUO group appeared to exhibit deficits in social interaction behaviors. In the literature, conflicting results exist regarding the impact of in utero and postnatal opioid exposure on social behaviors in animal models. In one study of prenatal exposure to buprenorphine, methadone, and morphine, social interactions in exposed rats were impaired, similar to what has been reported in human studies^62^. In contrast, other studies have shown that prenatal morphine exposure increased social behaviors and resulted in less social avoidance^63,64^. Differences in results may be due to different experimental designs, such as behavior chambers and scoring or the dosing schedule of the animals. For example, many reported behavioral tests for prenatal opioid exposure studies have tested social interaction and play behavior in open chambers where the animals can interact freely^62–64^. Our social preference and novelty tests used a three-chambered apparatus that did not allow for free interaction or play between the test rat and the cagemate or naive animal. As for treatment differences, both Hol et al. and Niesink et al. treated rats with morphine during the last week of gestation and showed increased play behavior in the offspring^63,64^. Najam and Panksepp, however, showed that early postnatal treatment causes a delay in achieving control levels of play behavior in rats^65^. Our treatment paradigms started prior to mating and continued until weaning (IUO) or started only after giving birth and continued until weaning (PNO). Alterations in rodent behavior post treatment may depend on the stage of development at which the offspring are exposed to treatment, and social problems resulting from intrauterine^49^ or adolescent^36^ exposure may resolve with age. Our social behavioral tests occurred during young adulthood (P60–65), so perhaps any existing social deficits returned to baseline by the time of testing. The antisocial behavior exhibited by the F2 IUO animals may be attributed to the downregulation of *Avp* highlighted in our NAc RNA-seq and post-validation data. AVP systems have been shown to modulate social behaviors in rats^66^, and blocking AVP receptors resulted in significantly decreased investigation of novel objects^67^. Therefore, the regulation of *Avp* and its effects on social behavior in the context of perinatal opioid exposure may be an important avenue of study for future work.

In addition to social behaviors, we investigated compulsive and anxiety-like behaviors using marble burying tests in both generations. In our study, both the PNO and IUO groups in F1 buried more marbles than the control group, suggesting the presence of anxiety-like and obsessive-compulsive phenotypes. This pattern persisted into the F2 generation, demonstrating intergenerational effects of maternal opioid exposure on subsequent generations. Importantly, the hypocretin system has been implicated in anxiety disorders^68^. Hcrtr1 antagonists have been shown to attenuate anxiety-like behaviors^69^. Further, Hcrtr1 activity is anxiogenic, while Hcrtr2 activity is anxiolytic^70^. The increased expression of *Hcrt* and *Hcrtr1* may contribute to the anxiety-like behaviors exhibited by both generations. Further, prenatal exposure to morphine and methadone has been shown to increase anxiety-like behaviors in light–dark transition and elevated plus-maze tests in both male and female rats^62^. Additionally, Rohbani et al. found that parental morphine exposure affected compulsive grooming and anxiety-like behaviors through marble burying in the offspring^71^. In the case of the F2 IUO, the F1 germ line that would produce F2 was directly exposed, contributing to the intergenerational effect we saw on anxiety-like behavior in this group. In a study using marble burying to assess autism-like and anxiety-like behaviors, increased marble burying occurred in F1, F2, and F3, suggesting that these behaviors may exist through transgenerational epigenetic inheritance^72^. Our F2 animals exhibited the same anxiety-like behaviors as the F1 animals; thus, extension of this study to F3 may elucidate whether transgenerational inheritance is involved in this behavioral phenotype. It is important to note that, while the number of marbles buried in the F1 oxy-exposed groups was roughly similar, the F2 IUO buried significantly more marbles than the F2 PNO, suggesting the IUO may be the more severely impacted of the two groups.

Overall, our study is the first to identify intergenerational effects of pre- and postnatal oxy exposure on development and behavior, thus prompting caution, as both routes of exposure can lead to developmental deficiencies that may persist into the next generation.

## Supplementary information

Supplementary Legends

Supplemental Statistical Data

Supplementary Figure 1

Supplementary Figure 2

Supplementary Figure 3

Supplementary Figure 4

Supplementary Table 1

Supplementary Table 2

Supplementary Table 3

Supplementary Table 4

Supplementary Table 5

Supplementary Table 6
