# Supplementary material for: Characterization of the intergenerational impact of in utero and postnatal oxycodone exposure
Source: Transl Psychiatry. 2020 Sep 23;10:329. doi: 10.1038/s41398-020-01012-z (PMC7511347; doi:10.1038/s41398-020-01012-z)
Supplement: Supplementary file 1 — Supplementary Legends [file 41398_2020_1012_MOESM1_ESM.docx]

**Supplementary Figure 1.** Schematic of dosing procedure for IUO and PNO dams and oxy exposure of F1 and F2 generations.

**Supplementary Table 1.** Complete list of animal numbers used in each group per experiment. Pups were also taken from these groups at different time points for other studies being done in parallel, hence the decrease in animal numbers over time.

**Supplementary Statistical Data.** Word document containing the variance values for each statistical test. The values are listed in order of each figure number, including supplementary.

**Supplementary Table 2.** Differential gene expression in the Nucleus Accumbens of PNO, IUO, and control animals in F1 and F2 generations. Group comparisons are split into separate tabs.

**Supplementary Figure 2.** RNA-seq analysis on P14 Nucleus Accumbens (NAc) of F1 and F2 animals. A) Clue-Go pie charts depicting affected biological processes in F1 and F2 generations resulting from differential gene expression between groups. Results are shown as the percent of genes per group. Asterisks represent the group term p-value of each category. **p < 0.001.

**Supplementary Table 3.** ClueGo analysis of biological processes affected by differential gene expression in the Nucleus Accumbens. Group comparisons are split into separate tabs.

**Supplementary Table 4.** Generational comparison of Nucleus Accumbens gene expression in saline and PNO groups of F1 and F2 generations. Common genes from group comparisons across the generations are listed in the “gene” tab.

**Supplementary Table 5.** Generational comparison of Nucleus Accumbens gene expression in saline and IUO groups of F1 and F2 generations. Common genes from group comparisons across the generations are listed in the “gene” tab.

**Supplementary Table 6.** Generational comparison of Nucleus Accumbens gene expression in IUO and PNO groups of F1 and F2 generations. Common genes from group comparisons across the generations are listed in the “gene” tab.

**Supplementary Figure 3.** A) Heatmap showing the complete list and expression of genes post-validated in F1 NAc. B) Heatmap showing the complete list and expression of genes post-validated in F2 NAc.

**Supplementary Figure 4.** A) During the social novelty test, F1 animals in each group had no significant differences in time spent in each chamber, number of chamber entries, or number of contacts with the naïve or cagemate animals. B) During the social preference test, F1 animals in each group had no significant differences in time spent in each chamber, number of chamber entries, or number of contacts with the naïve animal or toy.
