# Supplementary material for: Characterization of the intergenerational impact of in utero and postnatal oxycodone exposure
Source: Transl Psychiatry. 2020 Sep 23;10:329. doi: 10.1038/s41398-020-01012-z (PMC7511347; doi:10.1038/s41398-020-01012-z)
Supplement: Supplementary file 2 — Supplemental Statistical Data [file 41398_2020_1012_MOESM2_ESM.docx]

Supplemental Statistical Variances

Variances for each test were as follows.

**Figure 1A:** Body weight variances for each time point as assessed by two-way ANOVA were: P1 Generation (G): *F*_(1,398)_ = 0.1576, *P* = 0.6915; Treatment (T): *F*_(2,398)_ = 2.187, *P* = 0.1136; Interaction (I): *F*_(2,398)_ = 16.83, *P* < 0.0001; P7 G: *F*_(1,317)_ = 79.98, *P* < 0.0001; T: *F*_(2,317)_ = 1.445, *P* = 0.2373; I: *F*_(2,317)_ = 8.854, *P* = 0.0002; P14 G: *F*_(1,255)_ = 156.8, *P* < 0.0001; T: *F*_(2,255)_ = 0.6749, *P* = 0.5101; I: *F*_(2,255)_ = 19.22, *P* < 0.0001; P30 G: *F*_(1,151)_ = 41.50, *P* < 0.0001; T: *F*_(2,151)_ = 2.407, *P* = 0.0936; I: *F*_(2,151)_ = 21.03, *P* < 0.0001.

**Figure 1B:** Body length variances for each time point as assessed by two-way ANOVA were: P1 G: *F*_(1,390)_ = 42.05, *P* < 0.0001; T: *F*_(2,390)_ = 13.42, *P* < 0.0001; I: *F*_(2,390)_ = 5.077, *P* = 0.0067; P7 G: *F*_(1,322)_ = 3.119, *P* = 0.0784; T: *F*_(2,322)_ = 0.9494, *P* = 0.3881; I: *F*_(2,322)_ = 2.978, *P* = 0.0523; P14 G: *F*_(1,255)_ = 3.485, *P* = 0.0631; T: *F*_(2,255)_ = 1.615, *P* = 0.2009; I: *F*_(2,255)_ = 4.069, *P* = 0.0182; P30 G: *F*_(1,151)_ = 0.3937, *P* = 0.5313; T: *F*_(2,151)_ = 0.9907, *P* = 0.3737; I: *F*_(2,151)_ = 4.318, *P* = 0.0150.

**Figure 1C:** Head size circumference variances for each time point as assessed by two-way ANOVA were: P1 G: *F*_(1,390)_ = 52.68, *P* < 0.0001; T: *F*_(2,390)_ = 34.75, *P* < 0.0001; I: *F*_(2,390)_ = 3.774, *P* = 0.0238; P7 G: *F*_(1,322)_ = 28.16, *P* < 0.0001; T: *F*_(2,322)_ = 3.421, *P* = 0.0339; I: *F*_(2,322)_ = 0.1174, *P* = 0.8893; P14 G: *F*_(1,255)_ = 0.005627, *P* = 0.9403; T: *F*_(2,255)_ = 1.702, *P* = 0.1844; I: *F*_(2,255)_ = 1.038, *P* = 0.3556; P30 G: *F*_(1,151)_ = 19.36, *P* < 0.0001; T: *F*_(2,151)_ = 0.9286, *P* = 0.3973; I: *F*_(2,151)_ = 0.1185, *P* = 0.8883.

**Figure 2A:** BMI variances for each time point as assessed by two-way ANOVA were: P1 G: *F*_(1,390)_ = 73.48, *P* < 0.0001; T: *F*_(2,390)_ = 10.70, *P* < 0.0001; I: *F*_(2,390)_ = 7.846, *P* = 0.0005; P7 G: *F*_(1,322)_ = 55.74, *P* < 0.0001; T: *F*_(2,322)_ = 2.186, *P* = 0.1141; I: *F*_(2,322)_ = 2.548, *P* = 0.0799; P14 G: *F*_(1,255)_ = 78.02, *P* < 0.0001; T: *F*_(2,255)_ = 3.602, *P* = 0.0287; I: *F*_(2,255)_ = 2.688, *P* = 0.0699; P30 G: *F*_(1,151)_ = 26.58, *P* < 0.0001; T: *F*_(2,151)_ = 0.2094, *P* = 0.8113; I: *F*_(2,151)_ = 1.682, *P* = 0.1894.

**Figure 2B:** LOI variances for each time point as assessed by two-way ANOVA were: P1 G: *F*_(1,390)_ = 86.75, *P* < 0.0001; T: *F*_(2,390)_ = 15.51, *P* < 0.0001; I: *F*_(2,390)_ = 3.144, *P* = 0.0442; P7 G: *F*_(1,322)_ = 31.56, *P* < 0.0001; T: *F*_(2,322)_ = 1.936, *P* = 0.1459; I: *F*_(2,322)_ = 1.129, *P* = 0.3246; P14 G: *F*_(1,255)_ = 32.31, *P* < 0.0001; T: *F*_(2,255)_ = 3.346, *P* = 0.0368; I: *F*_(2,255)_ = 0.5747, *P* = 0.5636; P30 G: *F*_(1,151)_ = 16.14, *P* < 0.0001; T: *F*_(2,151)_ = 0.1138, *P* = 0.8925; I: *F*_(2,151)_ = 0.1001, *P* = 0.9048.

Because different genes were selected for post-validation in each generation, NAc genes were assessed separately in each generation by one-way ANOVA.

**Figure 3B:** F1 variances were: *Pmch* *F*_(2,7)_ = 203.8, *P* < 0.0001; *Hcrt* *F*_(2,7)_ = 7.502, *P* = 0.0182; *Otp* *F*_(2,7)_ = 5.603, *P* = 0.0352; *Avp* *F*_(2,7)_ = 26.43, *P* = 0.0005; *Cdh1* *F*_(2,7)_ = 57.70, *P* < 0.0001; *Tcf7l2* *F*_(2,7)_ = 5.563, *P* = 0.0358. F2 variances were: *Pmch* *F*_(2,14)_ = 25.60, *P* < 0.0001; *Hcrt* *F*_(2,14)_ = 4.099, *P* = 0.0397; *Avp* *F*_(2,14)_ = 40.38, *P* < 0.0001; *Hba1* *F*_(2,14)_ = 6.736, *P* = 0.0089; *Agrp* *F*_(2,14)_ = 7.297, *P* = 0.0067; *Aqp1* *F*_(2,14)_ = 10.51, *P* = 0.0016.

HCRT system gene expression between the generations was assessed with two-way ANOVA.

**Figure 3C:** *Hcrtr1* G: *F*_(1,21)_ = 4.022, *P* = 0.0580; T: *F*_(2,21)_ = 46.06, *P* < 0.0001; I: *F*_(2,21)_ = 2.342, *P* = 0.1208; *Hcrtr2* G: *F*_(1,25)_ = 0.1087, *P* = 0.7443; T: *F*_(2,25)_ = 0.6011, *P* = 0.5560; I: *F*_(2,25)_ = 1.323, *P* = 0.2843; *Nptx2* G: *F*_(1,23)_ = 0.08795, *P* = 0.7695; T: *F*_(2,23)_ = 56.78, *P* < 0.0001; I: *F*_(2,23)_ = 9.824, *P* = 0.0008; *Pdyn* G: *F*_(1,23)_ = 8.327, *P* = 0.0083; T: *F*_(2,23)_ = 5.499, *P* = 0.0112; I: *F*_(2,23)_ = 2.743, *P* = 0.0854.

Variances for social testing were assessed separately in each generation by two-way ANOVA.

**Supplementary Figure 4A:** F1 social novelty variances in time spent in each chamber were Chamber (C): *F*_(1,112)_ = 37.01, *P* < 0.0001; Treatment (T): *F*_(2,112)_ = 0.03170, *P* = 0.9688; Interaction (I): *F*_(2,112)_ = 1.014, *P* = 0.3660. Chamber entry variances were C: *F*_(1,112)_ = 0.6977, *P* < 0.4053; T: *F*_(2,112)_ = 3.284, *P* = 0.0411; I: *F*_(2,112)_ = 0.06349, *P* = 0.9385. Contact number variances were C: *F*_(1,112)_ = 24.34, *P* < 0.0001; T: *F*_(2,112)_ = 0.9495, *P* = 0.3900; I: *F*_(2,112)_ = 0.3385, *P* = 0.7135.

**Supplementary Figure 4B:** F1 social preference variances in time spent in each chamber were Chamber (C): *F*_(1,168)_ = 12.34, *P* = 0.0006; Treatment (T): *F*_(2,168)_ = 0.1620, *P* = 0.8506; Interaction (I): *F*_(2,168)_ = 0.9647, *P* = 0.3832. Chamber entry variances were C: *F*_(1,168)_ = 0.04244, *P* = 0.8370; T: *F*_(2,168)_ = 1.835, *P* = 0.1628; I: *F*_(2,168)_ = 0.04769, *P* = 0.9534. Contact number variances were C: *F*_(1,168)_ = 27.38, *P* < 0.0001; T: *F*_(2,168)_ = 0.2952, *P* = 0.7448; I: *F*_(2,168)_ = 0.3294, *P* = 0.7198.

**Figure 4A:** F2 social novelty variances in time spent in each chamber were C: *F*_(1,116)_ = 51.82, *P* < 0.0001; T: *F*_(2,116)_ = 1.285, *P* = 0.2806; I: *F*_(2,116)_ = 15.03, *P* < 0.0001. Chamber entry variances were C: *F*_(1,116)_ = 0.3805, *P* = 0.5385; T: *F*_(2,116)_ = 0.008232, *P* = 0.9918; I: *F*_(2,116)_ = 1.221, *P* = 0.2987. Contact number variances were C: *F*_(1,116)_ = 38.99, *P* < 0.0001; T: *F*_(2,116)_ = 3.525, *P* = 0.0326; I: *F*_(2,116)_ = 6.893, *P* = 0.0015.

**Figure 4B:** F2 social preference variances in time spent in each chamber were C: *F*_(1,120)_ = 49.30, *P* < 0.0001; T: *F*_(2,120)_ = 1.551, *P* = 0.2162; I: *F*_(2,120)_ = 3.107, *P* = 0.0484. Chamber entry variances were C: *F*_(1,120)_ = 2.040, *P* = 0.1558; T: *F*_(2,120)_ = 0.4941, *P* = 0.6114; I: *F*_(2,120)_ = 0.4373, *P* = 0.6468. Contact number variances were C: *F*_(1,120)_ = 45.34, *P* < 0.0001; T: *F*_(2,120)_ = 1.393, *P* = 0.2523; I: *F*_(2,120)_ = 2.126, *P* = 0.1238.

**Figure 4C:** Lastly, the variances for marble burying as assessed by two-way ANOVA were Generation: *F*_(1,179)_ = 20.25, *P* < 0.0001; Treatment: *F*_(2,179)_ = 37.05, *P* < 0.0001; Interaction: *F*_(2,179)_ = 8.404, *P* = 0.0003.
