# Supplementary figures and images for: Characterization of the intergenerational impact of in utero and postnatal oxycodone exposure
Source: Transl Psychiatry. 2020 Sep 23;10:329. doi: 10.1038/s41398-020-01012-z (PMC7511347; doi:10.1038/s41398-020-01012-z)

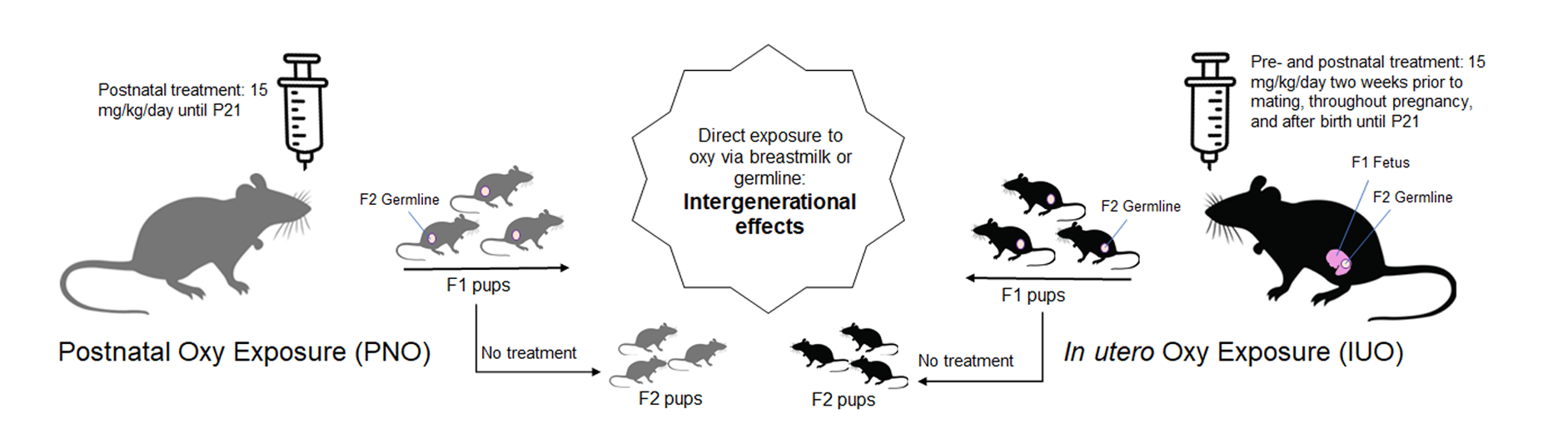

Supplement: Supplementary file 3 — Supplementary Figure 1 [file 41398_2020_1012_MOESM3_ESM.tif]

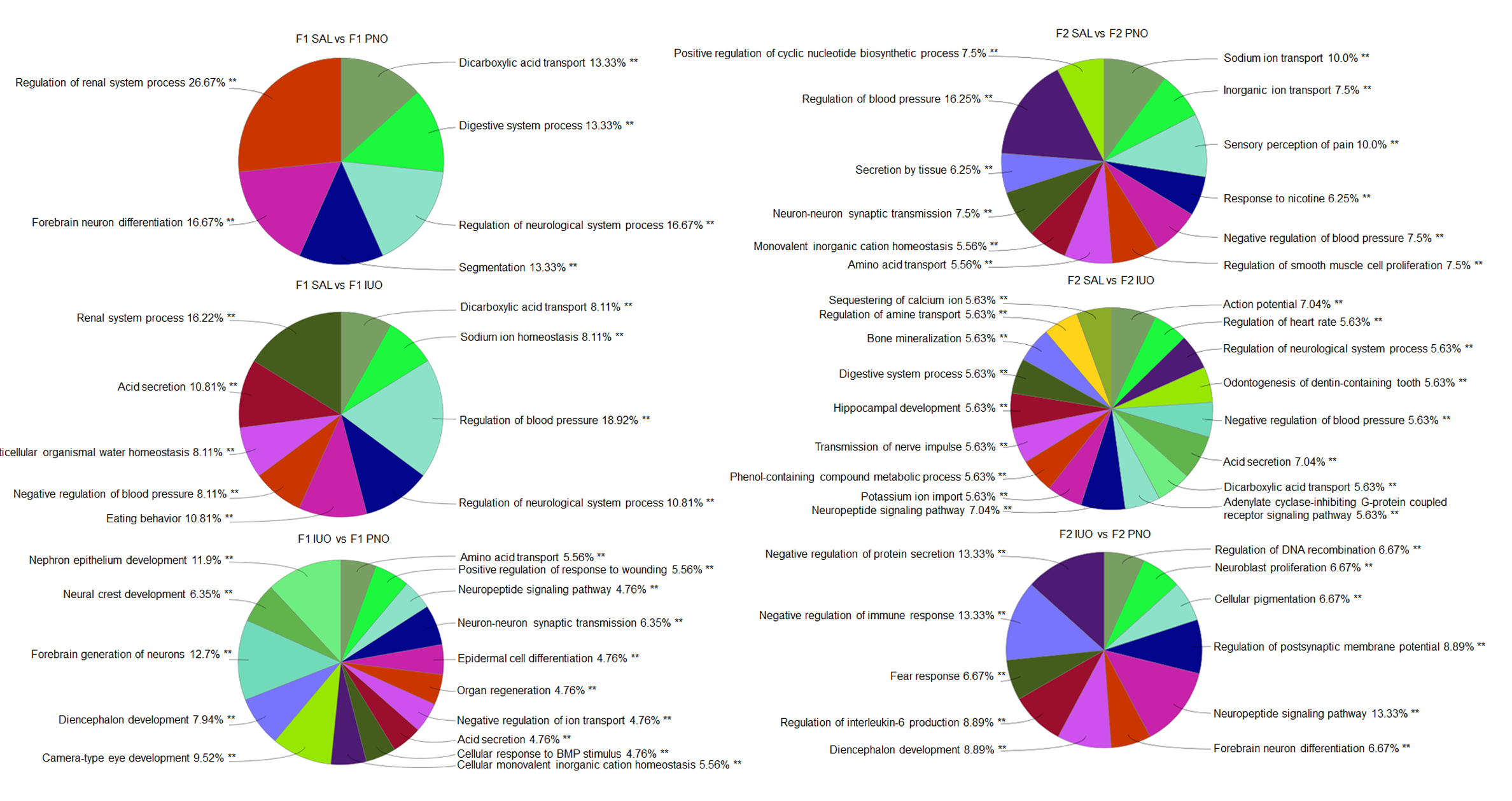

Supplement: Supplementary file 4 — Supplementary Figure 2 [file 41398_2020_1012_MOESM4_ESM.tif]

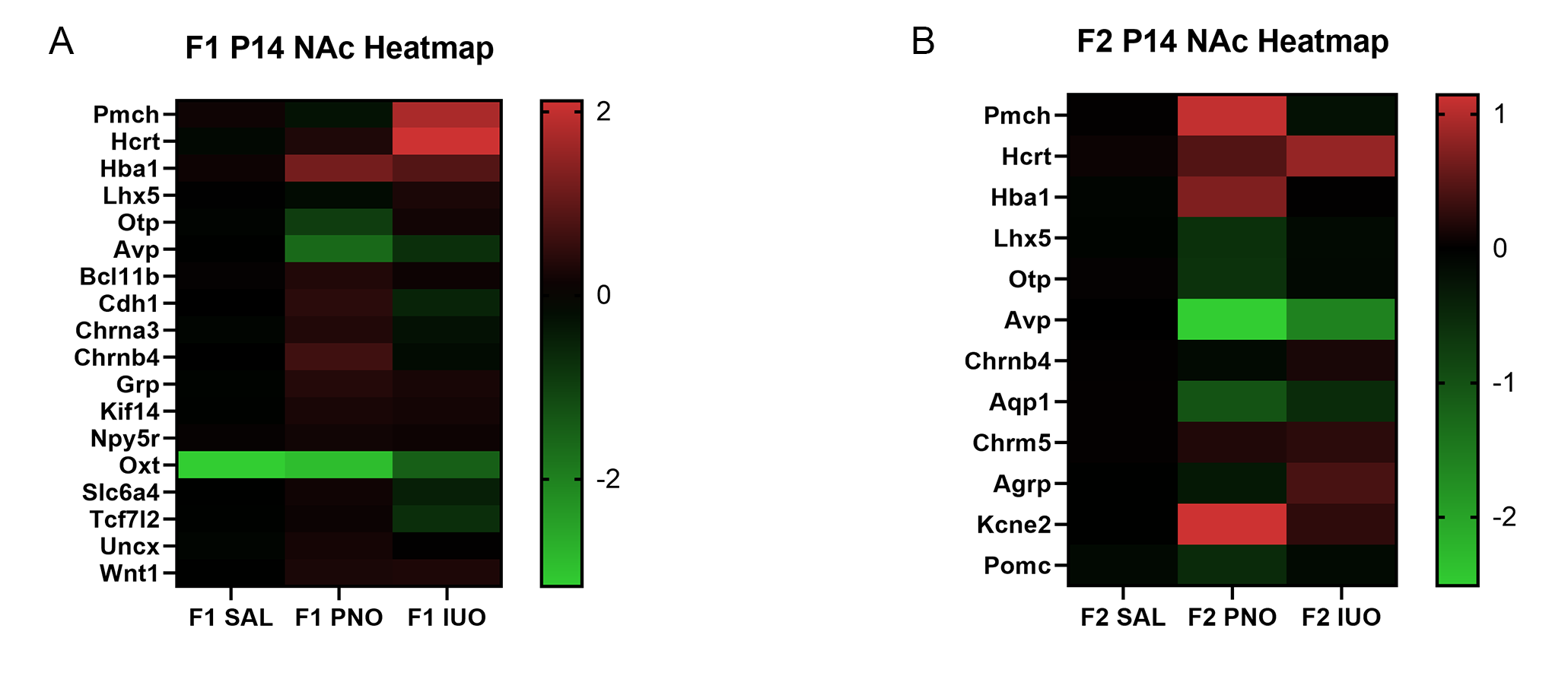

Supplement: Supplementary file 5 — Supplementary Figure 3 [file 41398_2020_1012_MOESM5_ESM.tif]

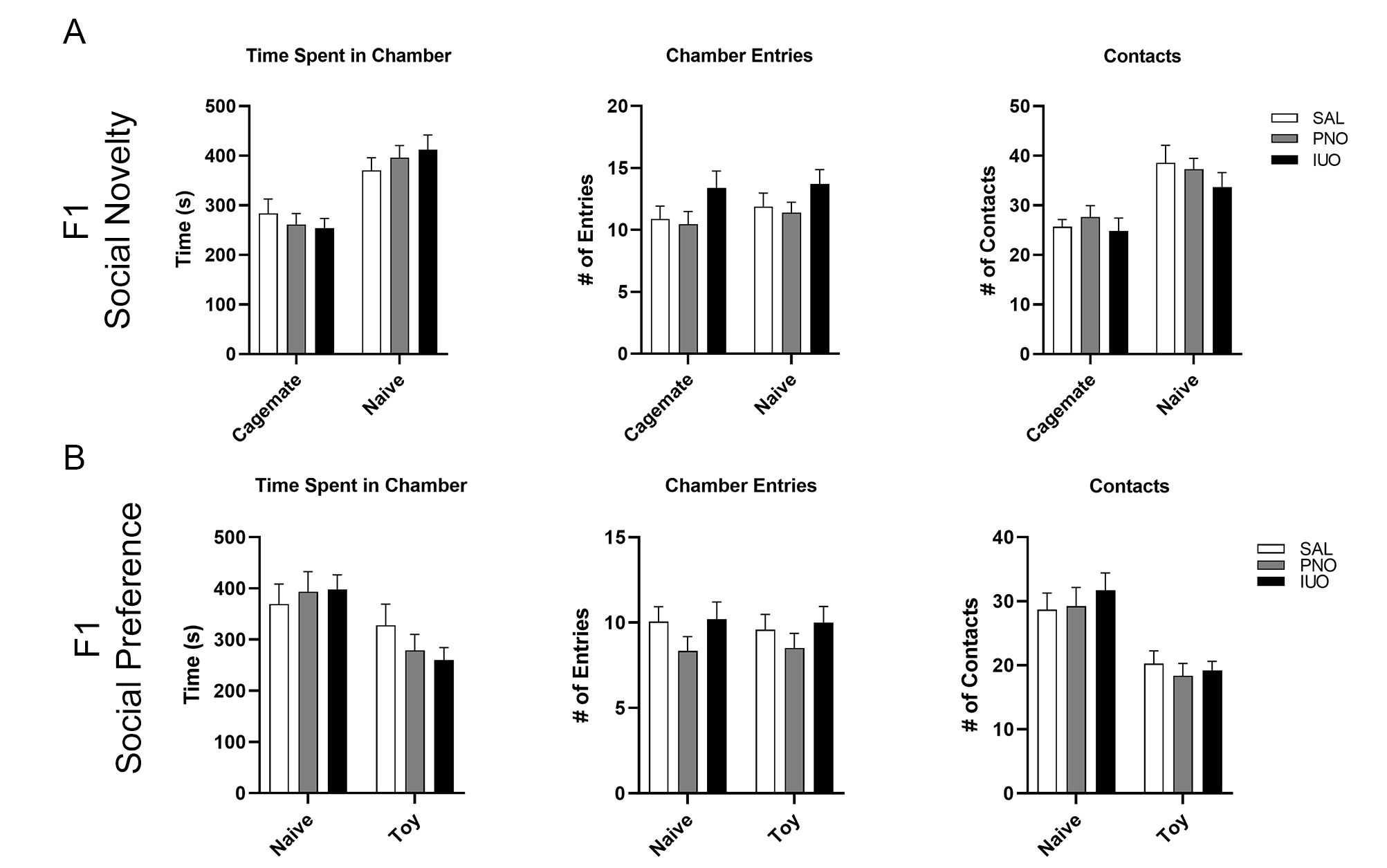

Supplement: Supplementary file 6 — Supplementary Figure 4 [file 41398_2020_1012_MOESM6_ESM.tif]
